# Association of abnormalities in electrocardiography and ultrasonic echocardiography with the occurrence of cardiovascular disease in patients with advanced chronic kidney disease

**DOI:** 10.1007/s10157-023-02437-8

**Published:** 2023-12-23

**Authors:** Ken Hirabayashi, Hideki Fujii, Keiji Kono, Satoshi Yamatani, Mao Shimizu, Kentaro Watanabe, Kazuo Sakamoto, Shunsuke Goto, Shinichi Nishi

**Affiliations:** Division of Nephrology and Kidney Center, Kobe University Graduate School of Medicine, 7-5-2, Kusunoki-cho, Chuo-ku, Kobe, Hyogo 650-0017 Japan

**Keywords:** Cardiovascular disease, Chronic kidney disease, Coronary artery disease, Electrocardiography, Ultrasonic echocardiography

## Abstract

**Background:**

In patients with chronic kidney disease (CKD), the incidence of cardiovascular disease (CVD) increases with disease progression. CVD screening tests in those with CKD were researched to determine whether abnormalities observed in electrocardiography (ECG) and ultrasonic echocardiography (UCG) were risk factors associated with the development of CVD.

**Methods:**

This study included 604 patients with CKD G4 and G5, for whom both ECG and UCG were performed. They were divided into four groups: those without ECG- and UCG-indicated abnormalities (group A, n = 333), with only ECG abnormalities (group B, n = 106), with only UCG abnormalities (group C, n = 75), and with both ECG and UCG abnormalities (group D, n = 90). Multivariate analysis using Cox regression analysis of the occurrence of CVD was performed during a follow-up period.

**Results:**

During the observation period, 124 patients had clinical events. Among them, 45 patients (13.5%) were in Group A, 25 patients (23.6%) in Group B, 19 patients (25.3%) in Group C, and 35 patients (38.9%) in Group D, respectively. CVD event occurrence was highest in Group D. The results of the multivariate analysis also showed that the CVD event rates were significantly higher in Group C (HR: 2.96, *P* = < .001) and D (HR: 4.22, *P* < .001) than in Group A.

**Conclusion:**

In patients with advanced CKD, there was a significant correlation of ECG and UCG abnormalities with CVD events. Additionally, those having both types of abnormalities may have a higher risk of coronary artery disease than other groups.

**Supplementary Information:**

The online version contains supplementary material available at 10.1007/s10157-023-02437-8.

## Introduction

Cardiovascular disease (CVD) is a critical complication and the leading cause of death in patients with chronic kidney disease (CKD) [1]. It has been reported that CVD accounts for approximately half of all deaths in this population [2]. In general, CVD includes coronary artery disease (CAD), heart failure (HF), and stroke (cerebral infarction and hemorrhage). Among them, CAD is particularly important because it is closely associated with HF, arrhythmia, and CVD mortality.

The prevalence of CAD is much higher in patients with CKD than in those without CKD, and the incidence of CVD increases along with the progression of CKD [3, 4]. Previous studies have reported that approximately 50–60% of patients with CKD already have CAD at the initiation of dialysis [5]. Furthermore, it is difficult to identify CAD among patients with CKD because their clinical symptoms and findings associated with CAD are often atypical [6]. Considering these facts, identifying CAD is clinically important, especially in patients with advanced CKD.

Screening tests for CAD are usually performed by stress electrocardiography (ECG), stress ultrasonic echocardiography (UCG), and stress myocardial scintigraphy in clinical settings. If the results suggest the presence of CAD, coronary angiography (CAG) is subsequently performed to definitively diagnose CAD. However, considering the cost, invasiveness, feasibility, and adverse effects; screening using simple tests is ideal. Examples include resting 12-lead ECG and UCG, which are commonly and easily performed in daily clinical practice. In addition, up to now, there are few studies that investigated the association between ECG and/or UCG abnormalities and CVD events in patients with advanced CKD [7–10].

Thus, the present study was designed and conducted to investigate the correlation of ECG and/or UCG abnormalities with CVD events and asymptomatic CAD in patients with advanced CKD.

## Methods

### Study design and population

This was a retrospective observational study that included 720 adult patients (all 20 years of age or older) with CKD stages G4 and G5, who were admitted at Kobe University Hospital between January 2010 and December 2019. Among them, 116 patients were excluded based on the following criteria: kidney transplant recipients (n = 25), newly diagnosed and treated glomerulonephritis (n = 78), pacemaker (n = 3), and insufficient clinical data (n = 10). The remaining 604 patients were enrolled in the study, and resting ECG and UCG were performed on all the study participants. Based on these cardiac examinations, we evaluated all suspicious findings of CAD (SFC) in each patient. Thereafter, the patients were divided into four groups depending on the presence of SFC as follows: Group A had no SFC in both cardiac examinations, Group B had SFC only indicated by ECG, Group C had SFC only indicated by UCG, and Group D had SFC indicated by both cardiac examinations. The differences in clinical characteristics, occurrence of cardiovascular events, and prognosis, were compared among the four groups. We followed up on each individual patient’s medical records until June 2020 to determine whether they had clinical events. As most patients attended our hospital as an outpatient, we could follow their outcomes. As for patients who transferred to other hospitals, we requested medical information to capture the follow-up data. Among them, for those without enough clinical information, we set the date and time when we were able to follow up on the patient's medical records at our hospital as the censoring date. Additional screening tests were performed based on the decision at a medical conference. Even after starting dialysis, we followed their clinical events.

This study was conducted in accordance with the principles stated in the Declaration of Helsinki. Our study protocol was approved by the appropriate institutional review committee (No. B210047). The ethical committee waived the need for informed consent in this study because the data were retrospectively and anonymously analyzed.

### Definition of SFC in the resting ECG and UCG

SFC in the resting ECG included ST depression, abnormal T waves (negative or flattened T waves), abnormal Q waves, or left bundle branch block [11]. Each ECG abnormality criterion, without left bundle branch block (LBBB), was defined according to the recommendations of the American Heart Association (AHA) [12] as one of the following: ST depression with horizontal or down-sloping ST-segment depression ≥ 1 mm at the J point in two or more adjacent leads, abnormal T waves with negative T wave (≥ 1 mm depression) or flattened T wave (≤ 1/10 the height of R-wave) in two or more consecutive leads, or abnormal Q-wave ≥ 1/3 the height of R-wave and ≥ 40 mV. As it is well known that LBBB is closely associated with CAD, we adopted it as one of the abnormal findings [13]. LBBB is defined as the following findings: either a QS or a small r wave with a large S wave in lead V1, and a notched R wave and no Q wave in lead V6 [14].

For the resting UCG, SFC was defined as left ventricular ejection fraction (LVEF) < 60% or asynergy of wall motion according to ACC/AHA 2006 guidelines [15]. LV wall motion was assessed by dividing the LV into 16 segments according to the guidelines of the American Society of Echocardiography [16].

### Additional screening tests for CAD

Stress ECG and stress myocardial scintigraphy were performed for 141 patients with strongly suspected CAD based on the determination of our conference. Stress ECG was performed on a treadmill or ergometer. Myocardial perfusion scintigraphy was performed during adenosine stress. The screening for CAD was considered positive when either test was positive.

### Definition of clinical outcomes

The occurrence of CVD events and all-cause mortality were evaluated during the observational period until June 2020. Non-fatal CVD events included stable angina pectoris, acute coronary syndrome (non-fatal myocardial infarction or unstable angina pectoris), HF, aortic dissection, aortic aneurysm, and stroke. CVD death was defined as sudden death and death due to CAD, HF, aortic dissection, aortic aneurysm, and stroke. CVD events included CVD death and non-fatal CVD events. Major adverse cardiovascular event (MACE) was defined as the composite of non-fatal CVD events and all-cause mortality.

### Statistical analysis

All statistical analyses were performed using IBM SPSS statistics software version 26.0 (SPSS, Inc., Chicago, IL, USA). Variables were presented as means ± standard deviation. When comparing clinical backgrounds among the four groups, χ^2^ test (for categorical variables) and one-way analysis of variance (for continuous variables) were used, followed by the Turkey’s HSD post-hoc test. The Kaplan–Meier method and log-rank test were performed to compare the outcomes between groups. Cox proportional hazard models were used to adjust for the confounders, including age, gender, a history of smoking, diabetes mellitus, a history of CVD, kidney function, and urinary protein. A *P* value of < 0.05 was considered statistically significant.

## Results

### Patients’ characteristics

**Fig. 1:**
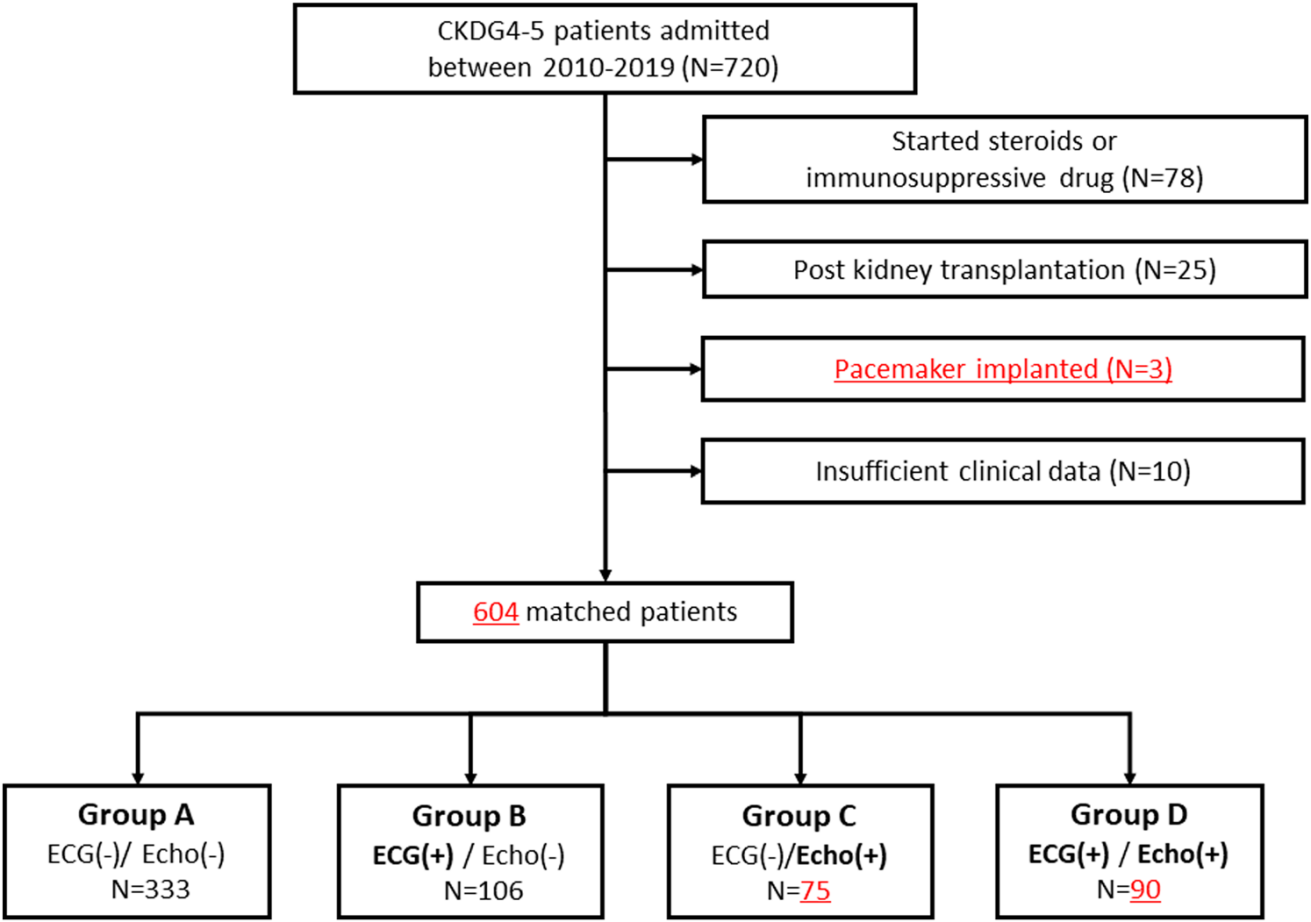
Enrollment flow of study patients

Study patients (n = 604) were divided into four groups as follows: Group A (n = 333), Group B (n = 106), Group C (n = 75), and Group D (n = 90) (Fig. 1). The median observational period was 21 months (quartile range 8–41 months). As for the clinical backgrounds, male gender, age, smoking, diabetes, a history of CAD and CVD, usage of beta-blockers and statins, uric acid levels, and BNP levels were significantly different among the four groups (Table 1). Group D had significantly more male patients and greater prevalence of histories of smoking, CAD, CVD, and usage of beta-blockers and statins, and significantly higher uric acid and BNP levels, compared to the other three groups. Furthermore, the prevalence of patients with diabetes was greater in groups C and D, compared to groups A and B.

**Table 1:** Patient characteristics

	Overall	Group A ECG (−) UCG (−)	Group B ECG (+) UCG (−)	Group C ECG (−) UCG (+)	Group D ECG (+) UCG (+)
	(N = 604)	(N = 333)	(N = 106)	(N = 75)	(N = 90)
Gender (male), n (%)	397 (65.7)	201 (60.4)	64 (60.4)	59 (78.7)^a,b^	73 (81.1)^a,b^
Age (y.o.)	65.5 ± 14.3	65.0 ± 14.5	69.4 ± 12.6 ^a^	61.3 ± 13.3^b^	66.3 ± 14.9
BMI (kg/m^2^)	24.3 ± 4.5	24.1 ± 4.4	23.7 ± 3.8	25.2 ± 5.5	24.4 ± 4.4
Smoking, n (%)	359 (59.4)	174 (52.3)	63 (59.4)	52 (69.3)^a^	70 (77.8)^a,b^
HT, n (%)	584 (96.7)	318 (95.5)	102 (96.2)	75 (100)	90 (100)
DM, n (%)	267 (44.2)	133 (39.9)	44 (41.5)	45 (60.0)^a,b^	45 (50.0)
DL, n (%)	475 (78.6)	253 (76.2)	84 (79.2)	65 (86.7)	73 (81.1)
CAD, n (%)	84 (13.9)	23 (6.9)	12 (11.3)	11 (14.7)^a^	38 (42.2)^a,b,c^
CVD, n (%)	192 (31.8)	82 (24.6)	36 (34.0)	23 (30.3)	52 (56.5)
CKD G4/G5, n (%)	222/382 (36.6/63.4)	123/210 (36.9/63.1)	42/64 (39.6/60.4)	26/49 (34.2/65.8)	31/59 (33.7/66.3)
RAS-I, n (%)	368 (61.0)	213 (64.0)	67 (63.2)	39 (52.6)	49 (54.3)
β-blockers, n (%)	184 (30.5)	81 (24.3)	36 (34.0)	20 (26.37)	47 (52.2)^a,b,c^
Statin, n (%)	263 (43.5)	135 (40.5)	50 (47.2)	34 (44.7)	44 (48.9)^a,b,c^
Anti-platelet agents, n (%)	173 (28.7)	76 (22.8)	34 (32.1)	19 (25.0)	44 (48.9)
eGFR (mL/min/1.73m^2^)	13.4 ± 7.3	13.3 ± 7.3	14.2 ± 7.4	12.5 ± 7.6	13.1 ± 6.8
UA (mg/dL)	7.3 ± 1.9	7.1 ± 1.8	7.4 ± 1.8	7.5 ± 1.8	7.9 ± 2.1^a^
Alb (g/dL)	3.5 ± 0.6	3.6 ± 0.6	3.4 ± 0.6	3.4 ± 0.6	3.5 ± 0.6
Proteinuria (g/gCr)	4.1 ± 3.8	3.8 ± 3.6	4.5 ± 4.5	4.4 ± 3.4	4.1 ± 3.9
BNP (pg/mL)	248.0 ± 455.9	129.0 ± 182.8	262.6 ± 330.8^a^	324.5 ± 486.1^a^	572.0 ± 871.0^a,b,c^
T-Chol (mg/dL)	182.6 ± 47.4	184.7 ± 49.9	185.2 ± 46.0	178.8 ± 49.3	174.8 ± 35.9
HDL-Chol (mg/dL)	53.9 ± 19.5	54.8 ± 19.8	55.8 ± 21.0	49.4 ± 16.8	51.9 ± 17.8
TG (mg/dL)	151.1 ± 76.9	151.3 ± 91.2	159.2 ± 89.9	157.6 ± 81.3	146.4 ± 70.6
LDL-Chol (mg/dL)	98.5 ± 38.9	100.1 ± 41.7	97.6 ± 34.4	98.6 ± 40.9	93.7 ± 31.0
HbA1c (%)	5.8 ± 0.8	5.7 ± 0.8	5.8 ± 0.8	5.8 ± 0.7	5.8 ± 0.8

*ECG* electrocardiography, *UCG* ultrasonic echocardiography, *BMI*, body mass index, *HT* hypertension, *DM* diabetes mellitus, *DL* dyslipidemia, *CAD* coronary artery disease; *CVD* cardiovascular disease, *CKD* chronic kidney disease, *RAS-I* renin–angiotensin–aldosterone inhibitor, eGFR estimated glomerular filtration rate, *UA* uric acid, *Alb* albumin, *BNP* brain natriuretic peptide, *T-Chol* total cholesterol, *HDL-Chol* high density lipoprotein cholesterol, *TG* triglyceride, *LDL-Chol* low density lipoprotein cholesterol, HbA1c hemoglobin A1c

Values are presented as the mean ± SD

^a^v.s. Group A, *P* < 0.05

^b^v.s. Group B, *P* < 0.05

^c^v.s. Group C, *P* < 0.05

### Cardiovascular disease events and death

**Fig. 2:**
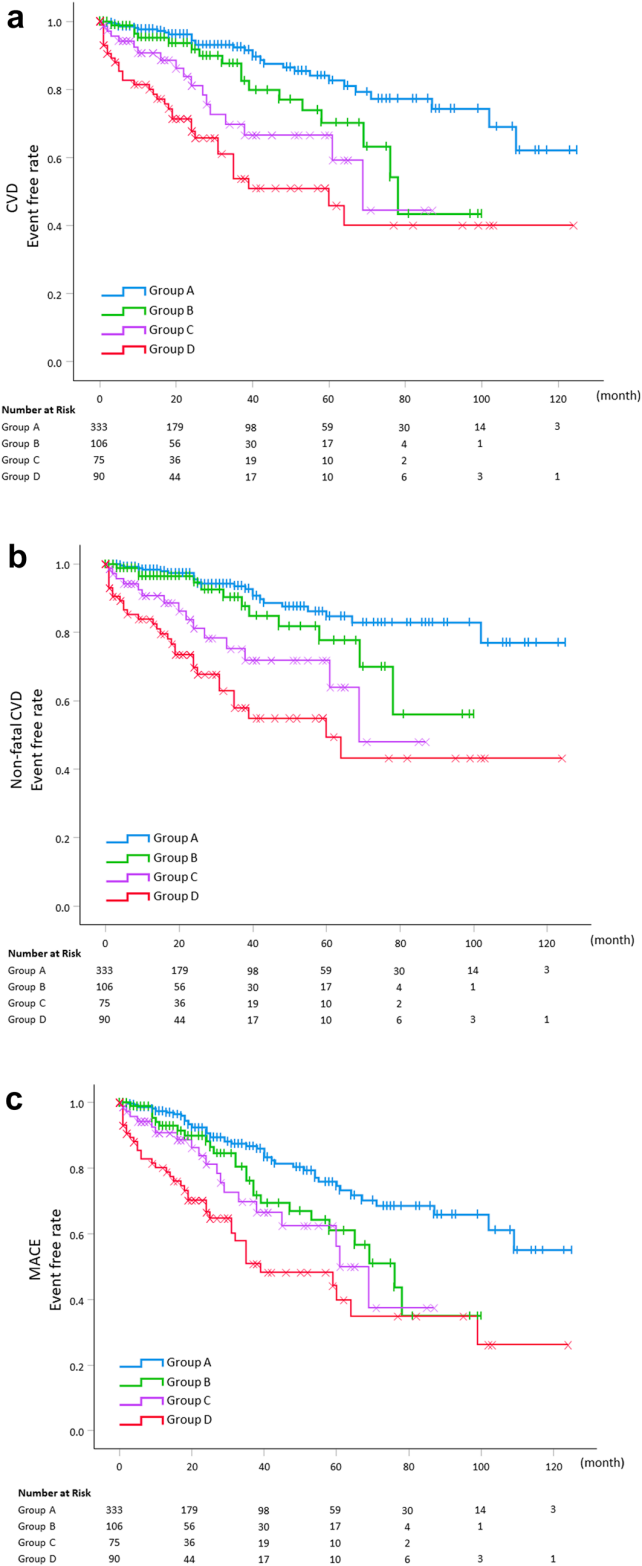
Occurrence of each clinical event among study patients according to findings of electrocardiogram and ultrasonic echocardiography. **a** CVD events. **b** Non-fatal CVD events. **c** MACE major adverse cardiovascular events. *CVD* cardiovascular disease, *MACE* major adverse cardiovascular events

During the observation period, 124 patients had some form of reportable event (Supplementary Table 1). Among 124 patients, 45 patients (13.5%) were in Group A, 25 patients (23.6%) in Group B, 19 patients (23.5%) in Group C, and 35 patients (38.9%) in Group D, respectively. The results of the Kaplan–Meier survival analysis showed that CVD events, non-fatal CVD events, and MACE were significantly greater in Group D compared to Group A (Fig. 2). Even after adjusting for covariates, such as gender, age, smoking, diabetes, and history of CAD, the hazard ratio for CVD events, non-fatal CVD events, and MACE remained significantly higher in Group C and D compared to Group A (Table 2). We analyzed the hazard ratios for each outcome by adjusting a history of CVD instead of a history of CAD, and these results were also similar (Supplementary Table 2). In addition, for adjustments of other covariates, we performed multivariate analysis by creating multiple models, and the results remained robust (Supplementary Table 3).

**Table 2:** Hazard ratio of each clinical event among study patients according to findings of electrocardiogram and ultrasonic echocardiography

	CVD				Non-fatal CVD				MACE			
	Unadjusted		Adjusted		Unadjusted		Adjusted		Unadjusted		Adjusted	
	HR (95% CI)	*P*	HR (95% CI)	*P*	HR (95% CI)	*P*	HR (95% CI)	*P*	HR (95% CI)	*P*	HR (95% CI)	*P*
Group A	Ref		Ref		Ref		Ref		Ref		Ref	
ECG (−)												
UCG (−)												
Group B	1.880 (1.006–3.513)	0.048	1.621 (0.884–2.971)	0.118	1.894 (0.913–3.929)	0.086	1.551 (0.760–3.165)	0.228	1.744 (1.043–2.916)	0.034	1.520 (0.924–2.499)	0.099
ECG (+)												
UCG (−)												
Group C	3.095 (1.695–5.651)	< 0.001	2.963 (1.587–5.533)	< 0.001	3.747 (1.929–7.279)	< 0.001	3.196 (1.609–6.349)	< 0.001	2.338 (1.360–4.018)	0.002	2.320 (1.321–4.074)	0.003
ECG ( −)												
UCG (+)												
Group D	4.007 (2.434–6.598)	< 0.001	4.223 (2.426–7.351)	< 0.001	4.912 (2.820–8.553)	< 0.001	5.098 (2.772–9.376)	< 0.001	3.660 (2.345–5.713)	< 0.001	3.011 (1.838–4.933)	< 0.001
ECG (+)												
UCG (+)												

*ECG* electrocardiography, *UCG* ultrasonic echocardiography, *CVD* cardiovascular disease, *MACE* major adverse cardiovascular event

### Prevalence of patients with positive screening tests for CAD

One hundred forty-one patients underwent screening tests for CAD, and 40 patients (28.4%) had positive results. The prevalence of patients with positive CAD screening results was 9.8% in Group A, 25.0% in Group B, 28.0% in Group C, and 56.8% in Group D, respectively (Supplementary Fig. 1). The prevalence was particularly high in Group D compared to the other groups.

## Discussion

The present study demonstrated that (1) patients with SFC as indicated by both ECG and UCG were significantly more likely to be male, and had a greater prevalence of a history of smoking, CAD, CVD, higher usage of beta-blockers and statins, and significantly higher uric acid and BNP levels compared to the other three groups. (2) CVD events, non-fatal CVD events, and MACE were significantly more prevalent in patients with SFC indicated by both ECG and UCG than in those without SFC. (3) After adjusting for covariates, such as gender, age, smoking, diabetes, and a history of CAD; the hazard ratio for CVD events, non-fatal CVD events, and MACE remained significantly higher in patients with SFC indicated only by UCG, and among those with SFC indicated by both ECG and UCG, than in those without SFC. (4) Finally, the prevalence of patients with positive results for CAD screening was particularly high in patients with SFC indicated by both ECG and UCG.

Patients with CKD have several prevalent risk factors for CVD, such as hypertension and diabetes mellitus, and CKD itself is also an independent risk factor for CVD [17, 18]. Although this study included only patients with CKD stages G4 and G5, the prevalence of hypertension was 96.7% and that of diabetes mellitus was 44.2%. A previous study reported that the occurrence of CVD events was a 2.8-fold increase in CKD stage G4 and a 3.4-fold increase in CKD stage G5 compared to a population with eGFR > 60 mL/min/1.73 m^2^ [19]. Japanese cohort studies have also shown an increase in CVD events in patients with CKD stages G4 and G5 compared to those with CKD stage G3a [20]. Cardiovascular-related deaths were similarly reported in a meta-analysis study with HRs of 2.57 and 6.38 for CKD stages G4 and G5, respectively, compared to patients with normal kidney function [21]. Corresponding with these results, 31.8% of the present study patients had a history of CVD, and the occurrence of all CVD and non-fatal CVD events was 15.9% and 12.9%, respectively, among them during the observational period. MACE was observed in 20.5% of these patients. Additionally, although none of the study patients had chest pain or discomfort upon admission, 42 patients (7.0%) had significant coronary artery stenosis requiring revascularization during an approximately two-year follow-up period. Japanese population-based epidemiological study reported that the prevalence of CAD was approximately 0.3% in men and approximately 0.1% in women [22]. This suggests a higher prevalence of CAD in patients with advanced CKD compared to the general population. From these findings, it is considered important to identify CVD in patients with CKD.

The ECG is a simple and useful non-invasive test for evaluating underlying cardiac conditions and is widely utilized in routine clinical practice. However, as a resting 12-lead ECG often shows normal findings in patients with stable angina pectoris [23] and ECG findings related to CAD are atypical, particularly in patients with CKD [24–26], its utility is limited when used alone to evaluate cardiac conditions. In addition to ECG, UCG is often performed in clinical settings. The presence of regional wall motion abnormalities identified through UCG is SFC and provides grounds for further detailed tests [27]. Low LVEF seen with resting UCG has been shown to be an important risk factor for mortality in CAD, HF, and CKD [28, 29]. However, it has been proposed that routine UCG at rest is inappropriate to use to detect CAD in the absence of typical angina symptoms and other clinical findings [25, 30, 31]. Additionally, considering characteristics of patients with CKD, such as a high prevalence of asymptomatic myocardial ischemia, background CVD progression due to cardio-renal involvement, and remarkable hemodynamic changes, particularly in advanced CKD and end stage kidney disease (ESKD), UCG evaluation alone may also be warranted in these populations.

Although a previous cohort study showed that normal ECG and UCG findings could identify subjects in whom CVD risk was low [32], there have been no studies examining the relationship of abnormal ECG and/or UCG findings with CVD. A previous study reported that ECG interval prolongations were associated with higher mortality in ESKD patients evaluated for kidney transplantation [9]. Furthermore, a study including Japanese patients with CKD showed that low ejection fraction and high left ventricular mass index were significantly associated with subsequent CVD [10]. Therefore, we believed that evaluation using both ECG and UCG must be useful, and this study evaluated CVD events and mortality based on ECG and/or UCG abnormalities. The group with ECG or UCG abnormalities, and with both ECG and UCG abnormalities, had higher event rates compared to the group without both abnormal ECG and UCG findings. As might be expected, more than 40% of patients with a history of CAD had both abnormalities, but the results of Cox regression analysis after adjustment by confounding factors, including a history of CAD, showed that the presence of both ECG and UCG abnormalities was a significant and independent risk factor for developing CVD events including not only CAD but also other CVD. From these findings, we believe that ECG and UCG are useful tests for CVD screening in patients with advanced CKD.

Although the diagnostic gold standard by which the presence and severity of CAD are assessed is invasive coronary angiography (CAG), it needs a contrast medium and may lead to worsening kidney function after examination. Therefore, although CAG should not be avoided for all patients with CKD, considering the risks and benefits, it should be performed appropriately. Additionally, clinical guidelines recommend noninvasive tests to search for CAD in asymptomatic patients without CKD [27, 33, 34]. Exercise stress ECG is a simple and available test and also has the advantage of being an indicator of exercise tolerance and has prognostic value [35]. Myocardial scintigraphy has also been one of the most important and common non-invasive diagnostic cardiac tests for CAD. It can detect the blood flow conditions of the heart muscle itself and predict culprit vessels. As mentioned previously, particularly in patients with advanced CKD, there is a need to lower the threshold for CAD screening and to narrow down patients with CAD using tools to identify high pretest probability. Therefore, it is important to examine the utility of inexpensive and noninvasive tests such as resting ECG and UCG. In the present study, we supposed that a combination of abnormal ECG and UCG findings, and subsequent detailed examinations by exercise stress ECG or myocardial scintigraphy, could detect the presence of CAD. Among 141 patients who received screening tests for CAD, 40 patients (28.4%) had positive findings. Those in the group with both ECG and UCG abnormality (group D) had a significantly higher positivity rate for ischemia (56.8%) than those in other groups (group A: 9.8%, group B: 25.0%, and group C: 28.0%, respectively). In this study, the diagnostic accuracy of the ischemic findings associated with the development of CVD events had a sensitivity of 68.3% and a specificity of 83.0%. Myocardial scintigraphy and exercise stress tests have been reported to have reduced accuracy in detecting obstructive CAD in CKD because they represent higher rates of both false-negative and false-positive tests [30]. Therefore, both ECG and UCG abnormalities appear to be very crucial findings suitable for predicting CVD events, and those with both ECG and UCG abnormality have a particularly high probability of having asymptomatic CAD. The results of this study found that patients with both ECG and UCG abnormality had a remarkably high CVD event rate compared to those without abnormal findings. This might be due to the fact that those with both abnormal findings frequently had CAD. Therefore, we believe that further detailed examination in order to detect CAD in such patients should be performed.

This study has several limitations. First, there is a possibility that we could not trace a few cases although most patients attended our hospital as outpatients and we got medical information from other hospitals. Second, as the additional screening tests for CAD were not performed for all the study patients, there could be a selection bias. Third, we might have omitted some important confounding factors that influence the results of the analysis because there is a limit to the number of confounders that can be adjusted for outcome based on the number of occurrences.

## Conclusion

In conclusion, in advanced CKD, ECG and UCG appear to be useful for the prediction of CVD. Additionally, in patients with both abnormalities, the prevalence of CAD may be particularly high.

### Supplementary Information

Below is the link to the electronic supplementary material.

Supplementary file1 (DOCX 40 KB)

Supplementary file2 (TIF 61 KB)

## Data Availability

The protocol of this study was registered in the University hospital Medical Information Network (UMIN). The registration number was UMIN000047684. Data underlying this study can be accessed through the UMIN repository system at: https://center6.umin.ac.jp/cgi-open-bin/icdr_e/ctr_view.cgi?recptno=R000054366.
