# Supplementary material for: Association of abnormalities in electrocardiography and ultrasonic echocardiography with the occurrence of cardiovascular disease in patients with advanced chronic kidney disease
Source: Clin Exp Nephrol. 2023 Dec 23;28(4):307–15. doi: 10.1007/s10157-023-02437-8 (PMC10954921; doi:10.1007/s10157-023-02437-8)
Supplement: Supplementary file 1 — Supplementary file1 (DOCX 40 KB) [file 10157_2023_2437_MOESM1_ESM.docx]

**Supplementary table 1. Type and prevalence of CVD and death**

|  | **All patients** | **Group A** | **Group B** | **Group C** | **Group D** |
| --- | --- | --- | --- | --- | --- |
| All events, n | 124 | 45 | 25 | 19 | 35 |
| ACS, n (%) | 17 (13.7) | 8 (17.8) | 1 (4.0) | 1 (5.3) | 7 (20.0) |
| CAD, n (%) | 25 (20.2) | 4 (8.9) | 4 (16.0) | 5 (26.3) | 12 (34.3) |
| Stroke, n (%) | 23 (18.5) | 10 (22.2) | 3 (12.0) | 6 (31.6) | 4 (11.4) |
| Hear failure, n (%) | 12 (9.7) | 0 (0) | 3 (12.0) | 3 (15.8) | 6 (17.1) |
| Aortic dissection, n (%) | 1 (0.8) | 0 (0) | 1 (4.0) | 0 (0) | 0 (0) |
| CVD death, n (%) | 18 (14.5) | 8 (17.7) | 5 (20.0) | 2 (10.5) | 3 (8.6) |
| Non-CVD death, n (%) | 28 (22.6) | 15 (33.3) | 8 (32.0) | 2 (10.5) | 3 (8.6) |

CVD, cardiovascular disease; ACS, acute coronary syndrome; CAD, coronary artery disease.

**Supplementary table 2. Hazard ratio of each clinical event among study patients according to findings of electrocardiogram and ultrasonic echocardiography by adjusting a history of CVD.**

|  | **CVD** | | | | **Non-fatal CVD** | | | | **MACE** | | | |
| --- | --- | --- | --- | --- | --- | --- | --- | --- | --- | --- | --- | --- |
|  | Unadjusted | | Adjusted | | Unadjusted | | Adjusted | | Unadjusted | | Adjusted | |
|  | HR (95%CI) | *P* | HR (95%CI) | *P* | HR (95%CI) | *P* | HR (95%CI) | *P* | HR (95%CI) | *P* | HR (95%CI) | *P* |
| **Group A**  ECG (-)  UCG (-) | Ref |  | Ref |  | Ref |  | Ref |  | Ref |  | Ref |  |
| **Group B**  **ECG (+)**  UCG (-) | 1.880  (1.006-3.513) | 0.048 | 1.493  (0.812-2.746) | 0.197 | 1.894  (0.913-3.929) | 0.086 | 1.451  (0.709-2.968) | 0.309 | 1.744  (1.043-2.916) | 0.034 | 1.429  (0.867-2.356) | 0.162 |
| **Group C**  ECG (-)  **UCG (+)** | 3.095  (1.695-5.651) | <0.001 | 2.853  (1.540-5.287) | <0.001 | 3.747  (1.929-7.279) | <0.001 | 3.027  (1.537-5.960) | 0.001 | 2.338  (1.360-4.018 | 0.002 | 2.235  (1.283-3.892) | 0.005 |
| **Group D**  **ECG (+)**  **UCG (+)** | 4.007  (2.434-6.598) | <0.001 | 3.708  (2.193-6.271) | <0.001 | 4.912  (2.820-8.553) | <0.001 | 4.431  (2.479-7.922) | <0.001 | 3.660  (2.345-5.713) | <0.001 | 2.714  (1.705-4.319) | <0.001 |

ECG, electrocardiography; UCG, ultrasonic echocardiography; CVD, cardiovascular disease; MACE, major adverse cardiovascular event.

Covariates: male, age, smoking history, diabetes, history of cardiovascular disease.

Among the covariates in Table 2, history of cardiovascular disease was replaced instead of coronary artery disease.

**Supplementary table 3. Multivariate analysis for each clinical event among study patients by creating multiple models.**

| **CVD** | **Model 1** | | **Model 2** | | **Model 3** | |
| --- | --- | --- | --- | --- | --- | --- |
|  | HR (95% CI) | P value | HR (95% CI) | P value | HR (95% CI) | P value |
| Group A | Ref. |  | Ref. |  | Ref. |  |
| Group B | 1.387  (0.735-2.619) | 0.312 | 1.264  (0.666-2.398) | 0.473 | 1.254  (0.662-2.376) | 0.488 |
| Group C | 2.976  (1.594-5.557) | <0.001 | 2.890  (1.558-5.359) | <0.001 | 3.252  (1.746-6.054) | <0.001 |
| Group D | 4.014  (2.318-6.951) | <0.001 | 3.603  (2.133-6.084) | <0.001 | 3.810  (2.203-6.592) | <0.001 |
| **Non-fatal CVD** | **Model 1** | | **Model 2** | | **Model 3** | |
|  | HR (95% CI) | P value | HR (95% CI) | P value | HR (95% CI) | P value |
| Group A | Ref. |  | Ref. |  | Ref. |  |
| Group B | 1.371  (0.656-2.866) | 0.401 | 1.273  (0.607-2.672) | 0.523 | 1.269  (0.606-2.660) | 0.528 |
| Group C | 3.227  (1.625-6.408) | <0.001 | 3.108  (1.577-6.126) | 0.001 | 3.606  (1.822-7.137) | <0.001 |
| Group D | 4.841  (2.648-8.850) | <0.001 | 4.361  (2.444-7.780) | <0.001 | 4.683  (2.562-8.562) | <0.001 |
| **MACE** | **Model 1** | | **Model 2** | | **Model 3** | |
|  | HR (95% CI) | P value | HR (95% CI) | P value | HR (95% CI) | P value |
| Group A | Ref. |  | Ref. |  | Ref. |  |
| Group B | 1.330  (0.786-2.249) | 0.288 | 1.255  (0.739-2.131) | 0.400 | 1.253  (0.739-2.127) | 0.402 |
| Group C | 2.396  (1.361-4.220) | 0.002 | 2.354  (1.346-4.116) | 0.003 | 2.550  (1.452-4.478) | 0.001 |
| Group D | 2.951  (1.807-4.819) | <0.001 | 2.780  (1.744-4.432) | <0.001 | 2.864  (1.754-4.677) | <0.001 |

ECG, electrocardiography; UCG, ultrasonic echocardiography; CVD, cardiovascular disease; MACE, major adverse cardiovascular event.

Covariates (Model 1): male, age, smoking history, diabetes, history of coronary artery disease, urinary protein-creatinine ratio.

Covariates (Model 2): male, age, smoking history, diabetes, history of cardiovascular disease, urinary protein-creatinine ratio.

Covariates (Model 3): male, age, smoking history, history of coronary artery disease, history of cardiovascular disease, urinary protein-creatinine ratio.
