# Supplementary figures and images for: Association of abnormalities in electrocardiography and ultrasonic echocardiography with the occurrence of cardiovascular disease in patients with advanced chronic kidney disease
Source: Clin Exp Nephrol. 2023 Dec 23;28(4):307–15. doi: 10.1007/s10157-023-02437-8 (PMC10954921; doi:10.1007/s10157-023-02437-8)

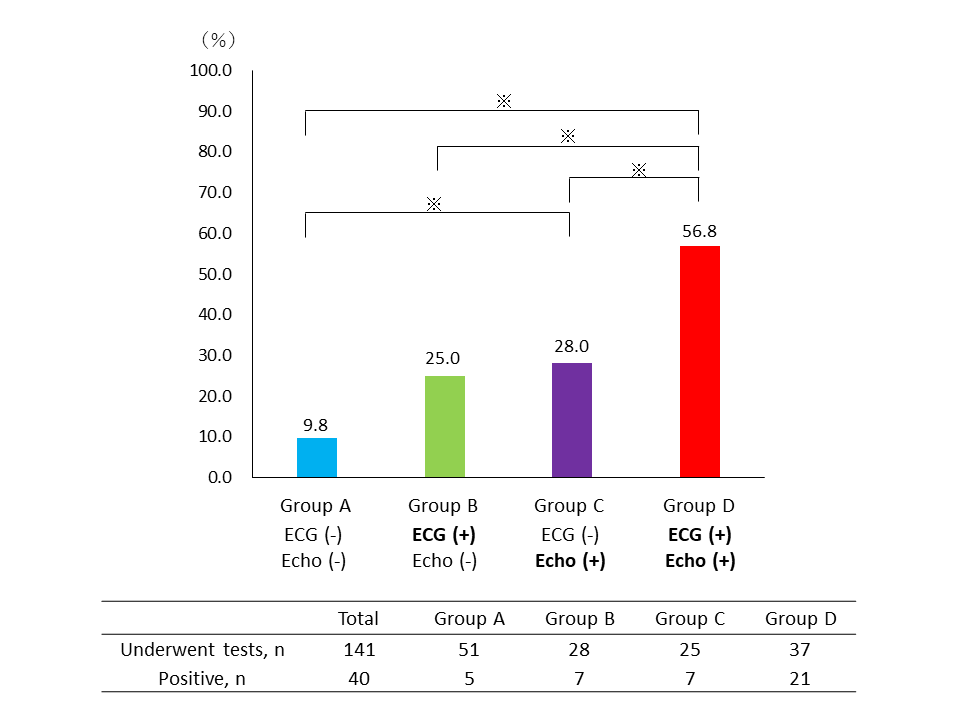

Supplement: Supplementary file 2 — Supplementary file2 (TIF 61 KB) [file 10157_2023_2437_MOESM2_ESM.tif]
